# Accuracy in Determining the Glycaemic Impact of Meals by Adding Individual Food Values Is Affected by Food Number, Homeostasis and Glucose Reference Dose

**DOI:** 10.3390/nu15153296

**Published:** 2023-07-25

**Authors:** John Monro

**Affiliations:** New Zealand Institute for Plant & Food Research, Palmerston North 4442, New Zealand; john.monro@plantandfood.co.nz; Tel.: +64-6-3556137

**Keywords:** glycaemic glucose equivalent, glycaemic load, virtual food component, glycaemia, food composition data

## Abstract

Summing glycaemic glucose equivalent (GGE) values of foods in a meal would be a practical way to predict the relative glycaemic impact (RGI) of the meal, without measuring the whole meal postprandial effect. However, as glycaemic response is non-linear, and glycaemic responsiveness per gram of glucose decreases with dose, addition accumulates inaccuracy. This research described determined inaccuracies accruing during addition of GGE values of foods and identifies approaches to reduce inaccuracy. By combining five published glucose dose–glycaemic response curves, the relationship between GGE dose and response was shown to be nearly quadratic (R^2^ = 0.98). This curve allowed determination of the divergence between the theoretically true glycaemic glucose equivalence of food intakes and estimates obtained by extrapolating linearly from zero through responses to glucose reference doses of 10, 20, 30, 40, 50 and 60 g. For each reference, the disparity between the linearly determined sum of GGE values of foods in 20 realistic meals, and true homeostasis-adjusted glucose equivalence for each whole meal, was calculated. Summation of the GGE values of individual foods could lead to inaccurate (>5 g GGE) estimates of the RGI of meals, depending on the GGE total, the number of foods, and the size of the glucose reference. Inaccuracy that accumulates during linear addition of GGE values of foods limits the range in which they can be used linearly in dietary management, public health and epidemiology. However, the steps discussed herein may be taken to allow for non-linearity.

## 1. Introduction

The effect of a quantity of food on a health end point or biomarker may be described in terms of a virtual food component (VFC)—a quantity that expresses the food effect as the equivalent weight of a reference of defined effect per unit weight [1]. VFCs are functional equivalents, so they express the relative impact of a food on a physiological state or outcome. They cannot state the actual effect until the responsiveness of an individual per unit of VFC has been established. However, once the sensitivity of a person per VFC is known, from their response to a reference food of known VFC content, their response to any other food of predetermined VFC content may be estimated. Thus, a potential clinical value of VFCs is that they would allow food combinations to be formulated within recommended limits imposed by a state of health, such as glucose intolerance. However, even when unable to predict an actual response, VFCs are intended to guide consumers to relatively healthier food choices.

As functional food attributes, VFCs may complement nutrient composition data to provide a more complete nutritional profile of a food than can be obtained from chemical analysis alone. Variables representing food effects are being increasingly incorporated into food composition databases to increase their value in dietary management for health, and to facilitate food choices based on efficacy [2,3]. However, a potential problem with functional food values (and nutrient values) is that when they represent outcomes that depend on physiological systems they may be subject to the effects of homeostasis. 

The glycaemic glucose equivalent (GGE) is a virtual food component that expresses the relative glycaemic impact (RGI) of a food, and is defined as the amount of glucose in grams that would induce the same glycaemic response as a given quantity of food [4,5]. As an equivalent, the GGE content of a food is ideally measured under equi-glycaemic conditions by using glucose references close to the GGE content of the food, or a standard curve, and realistic portion sizes [6,7].

The equi-glycaemic determination of GGE using customarily consumed portions is aimed at increasing relevance, and at providing a more accurate estimation of glucose equivalence than that obtained from the glycaemic index (GI), which is based on a 50 g glucose reference and 50 g available carbohydrate portion sizes. Because of homeostasis, the glycaemic response is very consistently an almost quadratic function of glucose dose [5], shown to reach a plateau (or maximum) between 60 and 100 g of glucose intake [7,8,9,10,11,12,13]. The glycaemic response per gram of glucose will, therefore, change with dose, and an error will arise when the glucose equivalent of a food portion is estimated from a response that differs substantially from the response to a 50 g glucose reference dose. Even with a food mass delivering 50 g carbohydrate, as in GI determination, unless the food has a GI of 100 or more, the glucose equivalent of the food will be lower on the dose–response curve than the glucose reference. Furthermore, if the effect of a serving rather than a 50 g carbohydrate dose is measured, it will often be very much lower on the dose–response curve than the response to 50 g of glucose.

Therefore, whether GGE values or glycaemic load (GL) values calculated as glycaemic index × carbohydrate content are used as measures of RGI, inaccuracy will inevitably arise as one extrapolates linearly as a function of food intake away from the GGE (or glucose) reference quantity. Nonetheless, in dietary management of food effects, it is most practical and “user-friendly” to be able to assume a linear relationship between food intake and effects in meals of recommended moderate size. This would allow the effect of varying intakes of a single food, or of combining different foods into a meal, to be determined in the same way as they are for nutrients—by simple proportion or linear summation. The effect of non-linearity of response is a general issue for VFCs and other functional food values that involve physiological effects subject to homeostatic feedback control, and may also affect epidemiological interpretations such as those based on GLs calculated from food intake data. 

The degree of error that may be accumulated by assuming that the effects of meals may be determined by simple summation of food values has not yet been fully explored with respect to RGI values. In this paper, a robust glucose–dose glycaemic response relationship, based on data published by this and other laboratories [7,8,9,10,11,12,13], is used to theoretically determine the degree of inaccuracy associated with linear summation of GGE values of foods in 20 realistic mixed meals containing a broad range of foods and nutrients. The aim is to determine whether, and under what conditions, values would need to be corrected for non-linearity arising from homeostasis.

## 2. Materials and Methods

### 2.1. Establishing a Glucose Dose–Glycaemic Response Curve and a Definition of GE_50_

**Figure 1:**
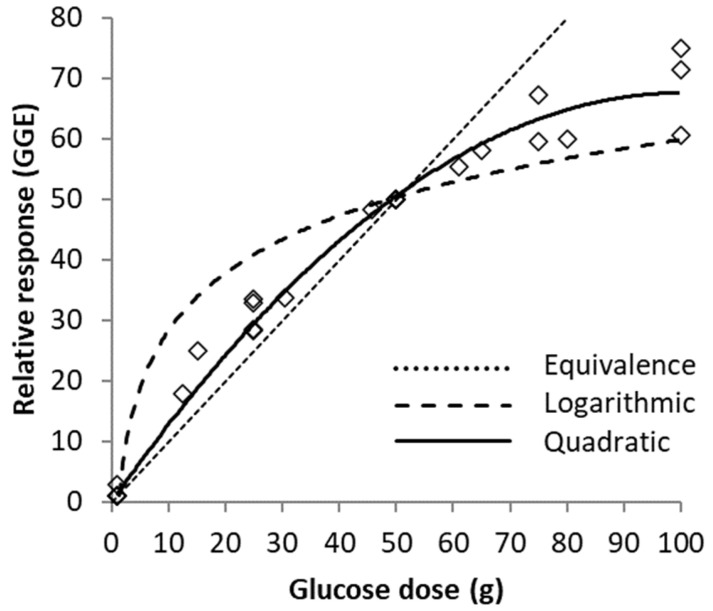
Glucose dose–glycaemic response relationship based on five published studies normalised to a relative response of 50 GGE at a glucose intake of 50 g with linear, polynomial, and logarithmic trendlines fitted. Linear (Equivalence): y = 0.692x + 9.005; R^2^ = 0.92. Quadratic: y = −0.006x^2^ + 1.35x; R^2^ = 0.98. Logarithmic: y = 13.69ln(x) − 3.23; R^2^ = 0.89.

A glucose dose–glycaemic response curve was established by combining published results from five independent studies of the relationship between glucose dose and glycaemic response [7,8,9,10,11,12,13]. The studies were all conducted under similar standardised conditions that allowed incremental changes in capillary blood glucose from a fasting state to be measured, followed by determination of the incremental area under the blood glucose response curve by trapezoid summation [14]. The results of each study were normalised by expressing them relative to the response to 50 g of glucose within each study, which was assigned a value of 50 GGE, and plotted against the glucose dose (Figure 1) [5]. 

In the case of glycaemic load, calculated from the glycaemic index, the unit of relative glycaemic response is one gram of glucose equivalent (GE) measured at an intake of 50 g of glucose, and extrapolated linearly to all carbohydrate intakes. In contrast, the glycaemic glucose equivalent (GGE) is the weight of glucose that would give the same glycaemic response as a specified mass of food. In addition, as glycaemic responsiveness changes with glucose dose, due to homeostasis, GGE is subject to dose, as is the accuracy with which GL represents glycaemic impact.

The present study explores how the number of units of relative response per gram of glucose changes, from 1 GE/g glucose at a 50 g glucose intake, as one moves from the 50 g anchor point to higher or lower intakes. The changes in GE (y-axis) per gram of glucose (x-axis) (Figure 1) is a measure of the dose-dependence of glycaemic responsiveness, and the equation of the relationship allows us to determine the effect of homeostasis and over the range of GGE intakes, and the inaccuracy associated with linear extrapolations from different reference points within the response range.

Comparison of polynomial, logarithmic, and linear trendlines, fitted to the data in Figure 1 using the Microsoft^®^ Excel^®^ trend fitting option, showed that a quadratic equation gave the best fit. A polynomial curve was fitted to the normalised five-study data and forced through zero (Figure 1), yielding the following equation:

y = −0.00670x^2^ + 1.346x; R^2^ = 0.98 (1)

where y is the relative response expressed as GGE and x is the glucose intake in grams. If not forced through zero, the equation was y = −0.00598x^2^ + 1.253x + 2.497. Equation (1) was used in the analysis as it was assumed that a zero response to zero food intake would make physiological sense, as discussed below.

### 2.2. Linear Extrapolations from Different Reference Responses and Their Difference from Quadratic

**Figure 2:**
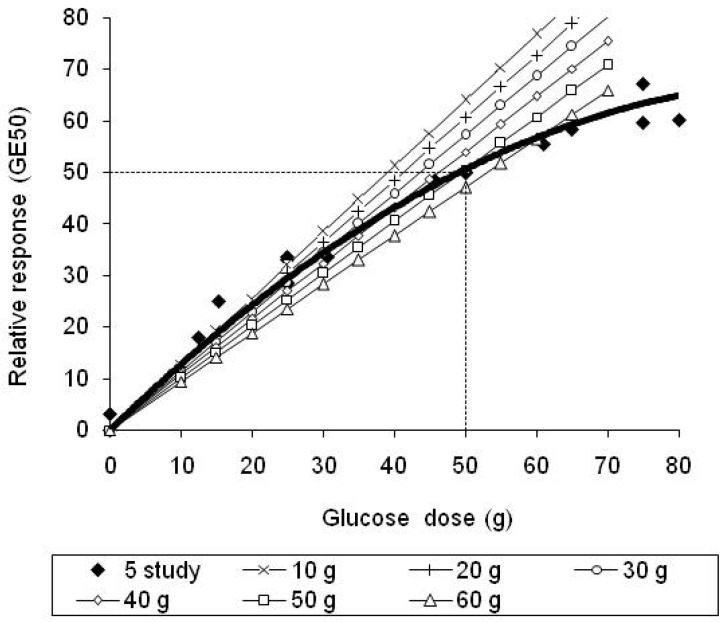
Linear extrapolations of relative glycaemic response to zero through 10, 20, 30, 40, 50 and 60 g glucose reference points on the quadratic glucose dose–relative glycaemic response curve.

Linear extrapolations from zero through responses to 10, 20, 30, 40, 50 and 60 g glucose references were achieved by entering the reference doses (x-axis values) into Equation (1) to obtain corresponding responses (y-axis), and then extrapolating from zero through the obtained value (Figure 2). Linear equations corresponding to the lines of extrapolation were obtained (Table 1). 

**Figure 3:**
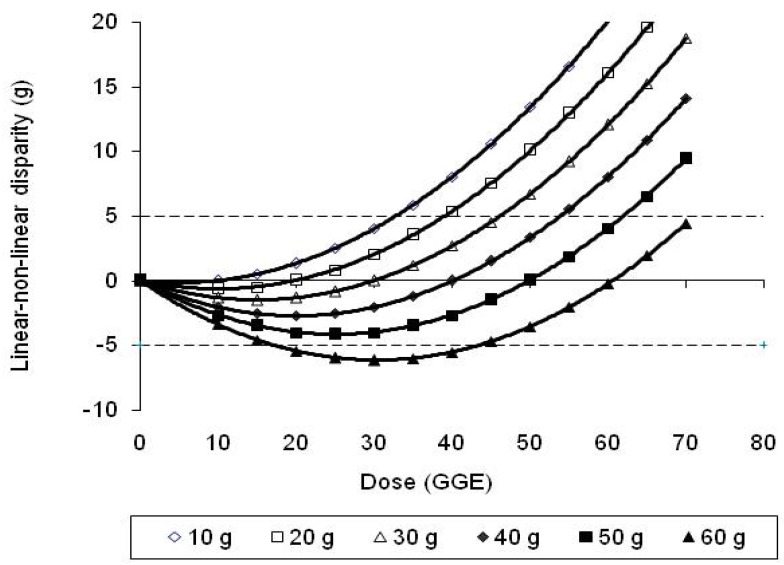
Disparities between true GGE on the quadratic glucose dose–glycaemic response curve and linear extrapolations from zero through glucose reference points, as a function of reference dose. Linear extrapolations underestimate true response between the reference and zero glucose intakes and is zero when the reference and GGE dose are matched.

Differences between the GGE values provided by Equation (1), which adjusts for dose–response curvature, and the values provided by the equations of linear extrapolation through each reference (Table 1), allowed the disparity between the linear and homeostasis-adjusted response curves to be calculated by subtraction. The discrepancies were plotted as a function of glucose intake (Figure 3), and polynomial trend lines fitted to yield the equations in Table 2.

### 2.3. Inaccuracy in Using Food GGEs Determined with a Single Reference to Obtain a Meal GGE

Foods comprising a series of composite breakfast meals, and which were referenced to the International table of glycaemic index and glycaemic load values: 2002 [15], were identified in a recent publication in which they were used as examples of realistic meals. The GL values (x) for the individual foods (Table 3) were calculated from the GI, available carbohydrate and portion size values (calculations not shown) [16], and used to calculate corresponding homeostasis-adjusted GGE values (y) using Equation (1). Several typical Australasian meals (Table 4) were also constructed using GL values and GE_50_ values and linear estimates calculated as for Table 3.

**Figure 4:**
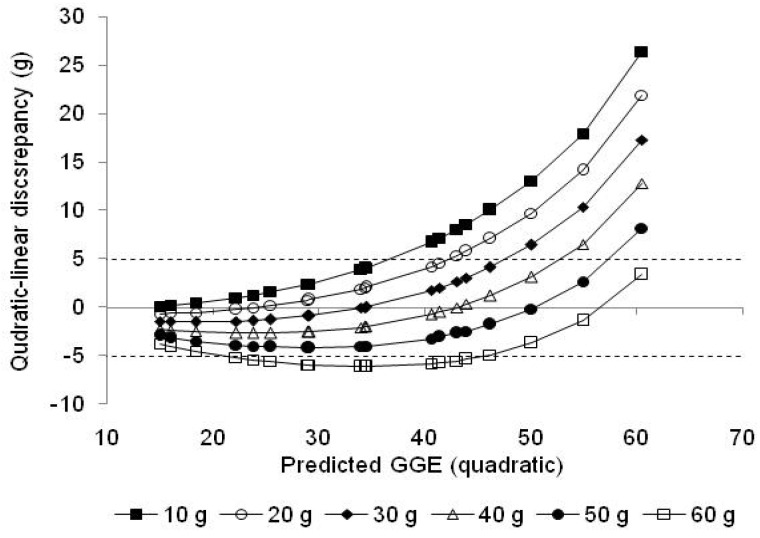
Discrepancies between quadratically determined GGE for whole meals, shown in Table 3 and Table 4, and the sum of linear estimates of individual food GGE values, as a function of the reference dose upon which the linear estimate was based.

**Figure 5:**
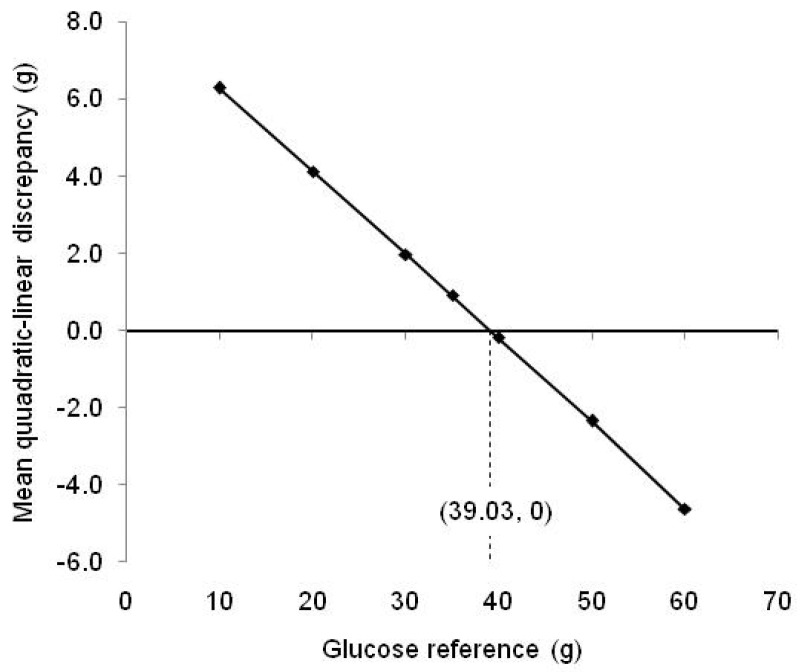
The dependence of the quadratic-linear discrepancy on the reference dose used shows that for meals 1–18 (less than about 50 GGE) in Table 3 and Table 4 a reference of 39.03 g glucose would have given a zero mean discrepancy.

The inaccuracy associated with within-meal summation of estimates of the GGE of individual foods, obtained using single glucose references of 10, 20, 30, 40, 50 and 60 g, was similarly determined by entering the GL values for the individual foods into the straight line equations in Table 1, adding the linear estimates of GGE for each food thus obtained, and subtracting them from the GGE value for the whole meal (Table 3 and Table 4). In this way, the discrepancy between the true whole meal GGE and meal GGE calculated from linear estimates of the individual foods in the meal was obtained for each meal (Figure 4). The average discrepancy between whole meal GGE values and summed meal GGE estimates for each glucose reference, for all 18 meals, was plotted against glucose reference dose to identify a reference giving the lowest mean discrepancy, that is, the greatest accuracy (Figure 5).

**Figure 6:**
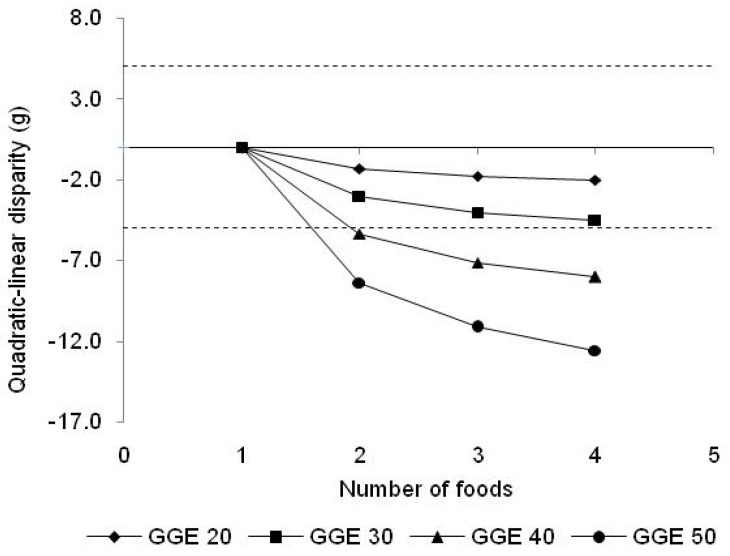
The effect of total GGE content of meal and number of carbohydrate foods on cumulative inaccuracy, assuming an equal distribution of GGEs between foods in a meal and using a glucose reference equal to the GGE content of the meal.

The theoretical dependence of cumulative inaccuracy on the GGE content of a meal, and on the number of contributing foods involved, was tested for 4 meals with total GLs of 20, 30, 40 and 50, with the totals distributed equally between 1, 2, 3 or 4 foods within each meal. True GGE for the whole meal was calculated using Equation (1), as well as the GGE for each food by linear extrapolation from the reference point; then, for each meal, the sum of the GGE values for the foods was added, and the sum total subtracted from the GGE for the whole meal, to obtain a value for the total of the inaccuracies of all of the foods within the meal (Table 5 or Figure 6).

### 2.4. Validity of Equation (1) Tested against Clinical Data

**Figure 7:**
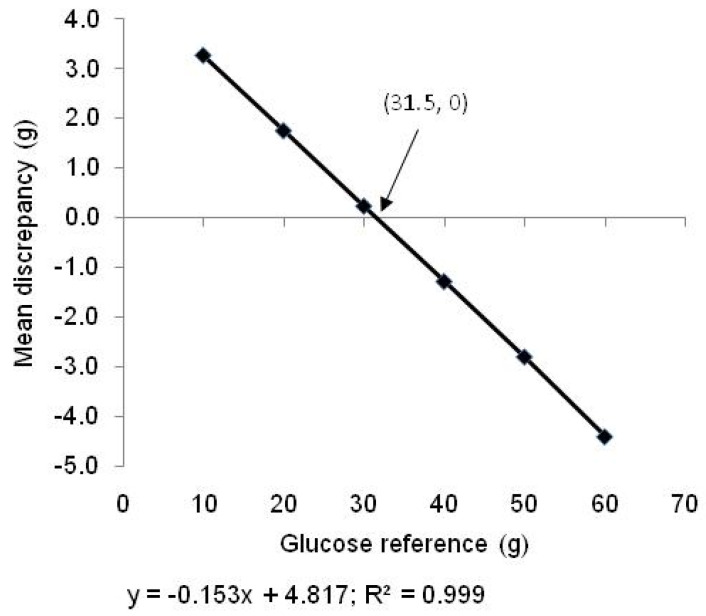
Effect of reference dose on the disparity between the mean of quadratic relative responses (GE_50_) and the mean of linear estimates (calculated from equations in Table 1) for a group of five foods × three intakes, published in Venn et al. [7]. Linear estimates coincided with quadratic values at a theoretical reference intake of 31.5 g glucose.

The validity of Equation (1) was tested for its ability to provide a correction for the disparity between GGE measured directly off a standard curve, and GL determined as the product of GI and available carbohydrate in the same experiment. GL is calculated as GI × carbohydrate, and GI is the ratio of the response to a 50 g carbohydrate portion of a food and the response to 50 g of glucose. However, when the response to 50 g of carbohydrate in a food is less than that to 50 g of glucose, the ratio will be affected by the non-linearity in the blood glucose response (Figure 1) and GL will overestimate GGE. If Equation (1) is valid, it should be able to be used to obtain GGE values from GL values by multiplying the GL values by the linear:quadratic ratio (L/Q). The L/Q correction was applied to data from an adequately powered clinical trial that we conducted [7], in which GL values were calculated from GI values determined within the trial, and in which GGE values were measured directly from a standard curve. A Bland–Altman analysis was conducted to determine whether the adjustment to GL using the L/Q ratio accurately predicted GGE (Figure 7).

**Figure 8:**
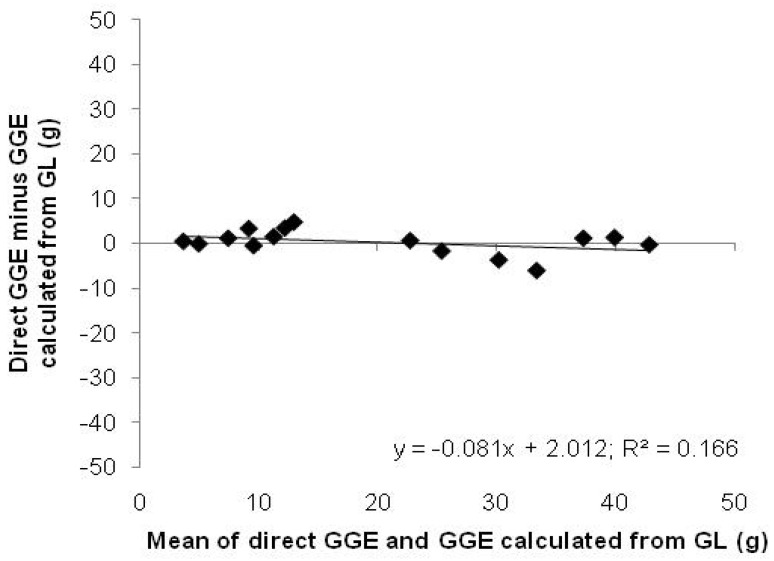
Bland–Altman analysis of GGE calculated as GL (the product of glycaemic index and carbohydrate) multiplied by the quadratic/linear ratio (from Figure 1), and of GGE measured directly.

The data from the clinical trial [7] also allowed us to determine a reference that would optimise accuracy for the group of foods used in the trial. The differences between the quadratic GGE values and the linear estimates based on the different references (Table 1) were calculated for each food and intake (i.e., 3 × 5 = 15 values). The mean quadratic–linear difference for each of the 15 food intakes was calculated, for each reference. This mean disparity for each reference was plotted against the reference doses to determine the reference dose that would give the greatest accuracy for the group of foods (Figure 8).

### 2.5. A Criterion Tolerable Discrepancy between True GGE and Linear Estimates

A tolerable disparity between true GGE represented by the quadratic response curve and linear estimates of GGE was calculated as the amount of dietary glucose that would raise blood glucose levels by 2 m·mol·L^−1^, from a fasting value of 5.0 m·mol·L^−1^ to 7.0 m·mol·L^−1^ defined as hyperglycaemia. An extracellular (plasma plus interstitial) fluid volume of 15 L in an average adult was assumed [17], a molecular weight of 180 g for glucose, complete transfer of dietary glucose to the extracellular fluids, and no metabolic and excretory losses or cellular uptake. Under such highly conservative theoretical conditions, the following holds:

Tolerable disparity (GGE) = (15 × 180 × 2)/1000 = 5.4 g ≈ 5.0 g glucose

### 2.6. Data Analysis

All calculations and graphs were conducted or produced in a standard Microsoft Excel spreadsheet. Equations were obtained from the trend lines inserted in the Excel chart option.

## 3. Results

The normalised dose–response curve (Figure 1) from zero to a plateau between 60 and 100 g glucose intake is the rising section of a standard inverted parabola and was better represented by a quadratic equation (R^2^ = 0.98) than a logarithmic curve (R^2^ = 0.89) in the 0–100 g region, in which most GGE intakes would lie (Figure 1). 

Because of the shape of the curve, any linear estimate of GGE as a relative response based on the response to a single glucose reference overestimated GGE by a small amount between zero and the point where the straight line estimate for each reference intersected the quadratic curve, but substantially underestimated it above the reference dose (Figure 2). The disparities between quadratic values and linear estimates based on all of the references were small in absolute terms for individual foods up to about 30 g of glucose or GGE intake (Figure 2), and the size of the disparity depended on the separation between the GGE content of the food and the size of the glucose reference.

However, when the GGE doses provided by individual foods were added, the linear sum often became sufficiently separated from the quadratic curve for the quadratic–linear difference to exceed the 5 GGE criterion of accuracy (Table 3 and Table 4, Figure 4). When GGE values were based on fixed glucose references, rather than on equiglycaemic determination, accuracy was quite well maintained if the reference was close to the total GGE for the meal as a whole. However, when the total GGE content of a meal was above 50 g, the use of single references of 10, 20 and 30 g of glucose led to large underestimations of the relative glycaemic impact. With the GGE (or GL) content of meals kept to less than 50 g glucose equivalents, a 30–40 g glucose reference gave greatest accuracy. Thus, with the two meals of highest glycaemic impact (GGE of 55 and 60.5 g) removed, the reference that would give zero average inaccuracy was about 39 g glucose (Figure 5).

The results in Table 3 and Table 4 showed that the greatest inaccuracies arose for meals of highest GGE content and containing more than one major source of carbohydrate, that is, more than one major source of inaccuracy. The dependence of cumulative inaccuracy on both the number of GGE sources and on the total GGE intake was confirmed in the results of Table 5 and Figure 6. The results showed that for meals of 30 GGE, cumulative inaccuracy increased with the number of foods involved but did not exceed 5 GGE, and that the 5 GGE threshold was exceeded for GGE intakes of 40 and 50 GGE.

The dependence of the disparity between GGE values measured off a standard curve (quadratic, Figure 1) compared with a single reference was confirmed with the clinical data of Venn et al. (Figure 7), from which a reference of 31.5 g was identified as giving the greatest accuracy with the group of foods used.

## 4. Discussion

The results showed that although homeostatic damping of response to food components may have a small effect on the accuracy of values representing effects of individual foods, it may have a more substantial effect on the accuracy with which individual food values may be added to determine a meal effect. The inaccuracy associated with linear addition of values for food effects suggests that the limits beyond which it is not valid to simply add individual food “functional” values to obtain a combined food effect should be identified.

The present paper explored the effect of homeostasis on values for the relative glycaemic effect of meals, by making use of the highly consistent finding that the intrinsic relationship between glucose intake and glycaemic response in humans is almost quadratic, between zero intake and a response plateau (maximum) that occurs in a glucose intake of between about 60 and 100 g. The analysis then applied the dose–response relationship to foods within a set of realistic meals, most of which (Table 3) have already been presented as being customarily consumed [16].

Although the quadratic equation (Equation (1)) describing the glucose dose–response relationship was the foundation for the present study, the relationship has also been described as logarithmic, but based on trials with small numbers of subjects [11]. It is evident from Figure 1, which is based on five studies combined, that the pattern of the blood glucose response to the glucose dose is highly consistent across studies and is well described by a quadratic equation in the dose range of interest. 

A largely theoretical analysis was considered necessary for this study because of the large variability associated with clinical measurements [18,19], which would make unmanageable the number of subjects required to adequately power clinical tests for the number of comparisons required [20]. However, the robustness of the dose–response relationship over a number of studies, and the fact that values from the only highly powered study of the dose–response relationship, that of Venn et al. [7] (60 (20 subjects × 3) measurements at 12.5, 25 and 75 g glucose and 80 (20 subjects × 4) at 50 g glucose dose), sits almost exactly on the combined curve, gives confidence that it may be used, as in this paper, to explore the inaccuracies that develop when RGI is assumed to be a linear function of carbohydrate intake. The non-linearity of the glucose dose–glycaemic response curve, and its repeatability, is to be expected of a specific physiological response that is under tight homeostatic control. 

The approach adopted is relevant because, in epidemiology and public health, differences that may not be easily measured as significant in single clinical studies, because of large individual variation, may be important to large populations over extended periods. Even at the individual level, where the moving average of effects over time is relevant to long-term outcomes, the analytical approach adopted in the present study has relevance.

The decision to force the multi-study curve (Figure 1) through zero requires comment, because the intercept of 2.5 GGE would have a significant bearing on the results if a tolerable inaccuracy is set at 5 GGE. The curve through zero was used for several reasons. Firstly, it made physiological sense to assume that in the absence of a stimulus there would be no response. Secondly, if a glycaemic response occurred from the expectation of food intake or from non-specific stimulation of the measurement situation, then it should be part of the baseline response for the effect of every food measured. Thirdly, there is no reason to believe that the response represented by the intercept would add to the response to an actual food intake, rather than be part of it.

The results showed for human data that, in general, the quadratic–linear disparity for individual foods is only a few GGE units (Figure 3), as has been shown in a clinical comparison of GGE values measured directly and GL values determined as GI × carbohydrate in the same experiment [7]. However, when directly measured GGE values are added together, the quadratic–linear disparities are also added and accentuated as the dose–response curve containing the reference point from which the linear estimate is made approaches a plateau, while the GGE summation continues to mount linearly. Thus, the sum of disparities of the individual food portions is greater than the disparity for the meal as a whole, and frequently exceeds the 5 GGE limit (Table 3 and Table 4, Figure 4).

If a single glucose reference is used to determine GGE indirectly, rather than a standard curve, the quadratic–linear disparity will be least when the GGE of the food portion and the reference dose are closely aligned [6]. The results of measuring the quadratic-linear disparities in the 20 meals in Table 3 and Table 4 suggest that if a single glucose reference dose is used, as in the measurement of glycaemic index, a reference of between 30 and 40 g of glucose would confer the greatest accuracy for general use, and would enable the GGE content of foods within meals to be safely added without adjustment, provided the meals contained a recommended moderate GGE content.

The results in Table 3 and Table 4 showed that the greatest inaccuracies arose for meals with the highest GGE content, over 30 GGE, containing more than one major source of carbohydrate, that is, containing more than one major source of inaccuracy. Such findings are to be expected, as the greater the number of sources of inaccuracy and the greater the GGE quantities involved, the greater the total inaccuracy. However, in the context of glycaemic control, an intake of 30 GGE may be a substantial intake of food or carbohydrate if the relative glycaemic potency (GGE/100 g food) of the food or the GI of the carbohydrate in it is low. For instance, in the study of Venn et al. [7], three granola bars (GI = 54) containing 78 g of available carbohydrate contributed 30.3 GGE, two servings of instant potato (GI = 90) containing 29.6 g available carbohydrate contributed 30.6 GGE, while one 70 g serving of cooked chickpeas (GI = 35) containing 12.9 g available carbohydrate contributed only 4 GGE. The importance of inaccuracies that develop when intakes of individual foods are far greater than the quantities used for GGE determination, or when GGE values for foods in a meal are added, should therefore be seen in the context of achieving glycaemic control through appropriate food choices. If recommendations for moderate food intakes and choice of carbohydrate foods of low relative GI are followed for glycaemic control, with use of appropriate references, inaccuracy in linear addition of GGE values should be easily minimised.

Clinical data that directly indicate an optimal reference size are at present limited, but data from a recent study allowed the mean quadratic–linear difference to be calculated with references of 10, 20, 30, 40, 50, and 60 g of glucose for each of 15 food intakes (Table 2 in Venn at al. [7]). When this mean difference for each reference was plotted against the reference dose, it showed that the disparity would be zero with a reference dose of 31.5 g of glucose (Figure 7). This supports the above suggestion, based on meals analysed in Table 3 and Table 4, that 30–40 g of glucose would be an appropriate single reference to use in obtaining RGI values for managing the postprandial glycaemic impact of mixed diets.

Food matrix effects that result from combining foods of different composition in meals were not part of the present analysis. However, in their analysis of the glycaemic responses to meals 1–13 (Table 4), Wolever et al. [16] could not find any evidence that fat or protein had a significant effect on the responses to the meals. Even where food matrix effects occur, however, the errors associated with summation of GGE values will remain because the homeostatic response is systemic and occurs after glucose absorption from the gut, where the primary food interactions affecting glycaemic response occur.

An advantage of using VFCs in blood glucose management is that they are relative rather than absolute values. Therefore, if there are components of a meal that suppress glycaemic response, such as when organic acids delay gastric emptying [21], the relativities and therefore the GGE content should remain constant. Foods of high glycaemic potency should still be able to be discriminated from foods of low glycaemic impact. So, the role of VFCs in guiding selection of relatively healthier food choices should be unaffected. In addition, if the response to a reference food under the conditions of a particular type of food matrix is known, the impact of blood glucose may be estimated. However, there seems to have been little research on the use of reference foods within different meal matrices. In practical management of glycaemia, the linear addition of GGE values for individual foods to obtain a meal RGI would be simplest, and as the present paper shows, may be used within limits. However, with modern electronic nutrient information systems being readily available, equations to adjust for non-linearity may be in-built, particularly as the linear–quadratic discrepancy appears to be a universal expression of human physiology, for which a universal mathematical adjustment should be possible. The accurate addition of food values would be easily achieved by converting all the non-linear values for carbohydrate foods in a meal to their corresponding linear (x-axis) values, using Equation (1), adding the linear values along the x-axis, and then converting the sum back to the non-linear “true” value. The close correspondence between GL values calculated from the glycaemic index and adjusted by the linear–quadratic disparity to give GGE values, and directly measured GGE values (Figure 8), suggests the mathematical adjustment is accurate.

The intrinsic non-linearity of the glucose dose–response relationship imposes limits on the use of relative measures of RGI of foods to determine the RGI of meals by simple linear summation, due to the accumulation of inaccuracy. The problem can be reduced by using a glucose reference close to the meal RGI, by limiting the number of foods involved and the total glycaemic loading they impose, or overcome by a mathematical correction. However, provided recommended intakes of carbohydrate foods are not exceeded, the inaccuracy is not usually sufficient to prevent the RGI of meals from being determined by simple summation of GGE values of foods. Hence, there is clearly need for caution in using them in dietary management and epidemiological interpretation in populations that typically consume large, highly glycaemic food portions.

## 5. Conclusions

The non-linearity of the carbohydrate dose–blood glucose response relationship imposed by homeostasis can lead to inaccuracy when determining meal glycaemic impact as GGE by simple addition of individual food values. The degree of inaccuracy depends on the following:

- The difference between the glycaemic impact of the meal and that of the glucose reference used to determine the GGE content of the food.
- The number of carbohydrate foods whose individual inaccuracies contribute to the total meal inaccuracy.

The inaccuracy could be practically reduced by using a glucose reference of about 40 g, rather than the 50 g dose currently used in glycaemic index determination. In practice, the amount of inaccuracy is unlikely to be enough to reduce the effectiveness of diabetes management based on GGE values as long as recommended moderate intakes of carbohydrate foods in a meal are adhered to.

## Figures and Tables

**Table 1:** Equations of straight lines from zero through blood glucose response (y) to glucose reference dose (x).

Glucose Reference (g)	Linear Equations Linking Zero to Reference Response
10	y = 1.279x
20	y = 1.212x
30	y = 1.145x
40	y = 1.078x
50	y = 1.011x
60	y = 0.940x

**Table 2:** Equations for discrepancies between quadratic (homeostasis-adjusted) and linear estimates of GGE based on different glucose references.

Glucose Reference (g)	Equation of Disparity between Linear and True (Homeostasis-Adjusted) GGE
10	y = −0.0067x^2^ + 0.067x
20	y = −0.0067x^2^ + 0.134x
30	y = −0.0067x^2^ + 0.201x
40	y = −0.0067x^2^ + 0.268x
50	y = −0.0067x^2^ + 0.335x
60	y = −0.0067x^2^ + 0.4060x

**Table 3:** Accumulation of disparities between true and linear estimates of GGE when adding estimated GGE values for individual foods in a meal. Meals shown are realistic breakfast meals [16].

			Linear Estimates of GGE Using Equations in Table 1					
			Glucose Reference					
Diet	GL ^1^	GGE_50_^2^	10	20	30	40	50	60
1								
45 g fibre and fruit cereal	20.80	25.1	26.6	25.2	23.8	22.4	21.0	19.6
120 mL 1.4%-fat milk	1.90	2.5	2.4	2.3	2.2	2.0	1.9	1.8
80 g Rockmelon	2.60	3.5	3.3	3.2	3.0	2.8	2.6	2.4
80 g Pineapple	4.40	5.8	5.6	5.3	5.0	4.7	4.4	4.1
A Sum of food values	29.70	36.9	38.0	36.0	34.0	32.0	30.0	27.9
B ^3^ Whole meal GGE		34.07						
A-B (inaccuracy)		2.8	3.9	1.9	−0.1	−2.1	−4.0	−6.2
2								
45 g High-fibre cereal	6.70	8.7	8.6	8.1	7.7	7.2	6.8	6.3
120 mL 1.4%-fat milk	1.90	2.5	2.4	2.3	2.2	2.0	1.9	1.8
50 g Strawberries	0.50	0.7	0.6	0.6	0.6	0.5	0.5	0.5
100 g Grapefruit	2.30	3.1	2.9	2.8	2.6	2.5	2.3	2.2
A Sum of food values	11.40	15.0	14.6	13.8	13.1	12.3	11.5	10.7
B Whole meal GGE		14.47						
A-B (inaccuracy)		0.5	0.1	−0.7	−1.4	−2.2	−2.9	−3.8
3								
80 g Egg omelette	0.00	0.0	0.0	0.0	0.0	0.0	0.0	0.0
40 g Wholemeal bread	13.30	16.7	17.0	16.1	15.2	14.3	13.4	12.5
80 g Spinach	0.20	0.3	0.3	0.2	0.2	0.2	0.2	0.2
20 g Red capsicum	0.50	0.7	0.6	0.6	0.6	0.5	0.5	0.5
80 g Grilled tomato	0.80	1.1	1.0	1.0	0.9	0.9	0.8	0.8
A Sum of food values	14.80	18.7	18.9	17.9	16.9	16.0	15.0	13.9
B Whole meal GGE		18.45						
A-B (inaccuracy)		0.3	0.5	−0.5	−1.5	−2.5	−3.5	−4.5
4								
80 g Egg omelette	0.00	0.0	0.0	0.0	0.0	0.0	0.0	0.0
38 g Honey and oat-bran bread	11.20	14.2	14.3	13.6	12.8	12.1	11.3	10.5
80 g Spinach	0.20	0.3	0.3	0.2	0.2	0.2	0.2	0.2
20 g Red capsicum	0.50	0.7	0.6	0.6	0.6	0.5	0.5	0.5
80 Grilled tomato	0.80	1.1	1.0	1.0	0.9	0.9	0.8	0.8
A Sum of food values	12.70	16.2	16.2	15.4	14.5	13.7	12.8	11.9
B Whole meal GGE		16.01						
A-B (inaccuracy)		0.2	0.2	−0.6	−1.5	−2.3	−3.2	−4.1
5								
15 g Whole-wheat cereal	6.60	8.6	8.4	8.0	7.6	7.1	6.7	6.2
120 mL 1.4%-fat milk	1.90	2.5	2.4	2.3	2.2	2.0	1.9	1.8
4 g Brown sugar	1.90	2.5	2.4	2.3	2.2	2.0	1.9	1.8
90 g Banana	9.40	12.1	12.0	11.4	10.8	10.1	9.5	8.8
100 mL Orange juice	4.90	6.4	6.3	5.9	5.6	5.3	5.0	4.6
A Sum of food values	24.70	32.2	31.6	29.9	28.3	26.6	25.0	23.2
B Whole meal GGE		29.16						
A-B (inaccuracy)		3.0	2.4	0.8	−0.9	−2.5	−4.2	−5.9
6								
64 g Muffin	19.60	23.8	25.1	23.8	22.4	21.1	19.8	18.4
14 g Peanut butter	0.00	0.0	0.0	0.0	0.0	0.0	0.0	0.0
A Sum of food values	19.60	23.8	25.1	23.8	22.4	21.1	19.8	18.4
B Whole meal GGE		23.81						
A-B (inaccuracy)		0.0	1.3	−0.1	−1.4	−2.7	−4.0	−5.4
7								
30 g Corn flakes	21.10	25.4	27.0	25.6	24.2	22.7	21.3	19.8
125 mL 2%-fat milk	2.10	2.8	2.7	2.5	2.4	2.3	2.1	2.0
10 g Brown sugar	4.80	6.3	6.1	5.8	5.5	5.2	4.9	4.5
50 g Whole-wheat bread	15.40	19.1	19.7	18.7	17.6	16.6	15.6	14.5
10 g Margarine	0.00	0.0	0.0	0.0	0.0	0.0	0.0	0.0
125 mL Orange juice	6.00	7.8	7.7	7.3	6.9	6.5	6.1	5.6
A Sum of food values	49.40	61.5	63.2	59.9	56.6	53.3	49.9	46.4
B Whole meal GGE		50.14						
A-B (inaccuracy)		11.4	13.0	9.7	6.4	3.1	−0.2	−3.7
8								
110 g Twelve-grain bagel	39.60	42.8	50.6	48.0	45.3	42.7	40.0	37.2
30 g Cream cheese	0.50	0.7	0.6	0.6	0.6	0.5	0.5	0.5
125 mL Orange juice	6.00	7.8	7.7	7.3	6.9	6.5	6.1	5.6
A Sum of food values	46.10	51.3	59.0	55.9	52.8	49.7	46.6	43.3
B Whole meal GGE	47.81	51.42						
A-B (inaccuracy)		3.5	11.2	8.1	5.0	1.9	−1.2	−4.5
9								
50 g Whole-rye pumpernickel	10.00	12.8	12.8	12.1	11.5	10.8	10.1	9.4
10 g Margarine	0.00	0.0	0.0	0.0	0.0	0.0	0.0	0.0
45 g Cracked wheat cereal	16.20	20.0	20.7	19.6	18.5	17.5	16.4	15.2
10 g Brown sugar	6.80	8.8	8.7	8.2	7.8	7.3	6.9	6.4
125 mL 2%-fat milk	2.10	2.8	2.7	2.5	2.4	2.3	2.1	2.0
125 mL Orange juice	6.00	7.8	7.7	7.3	6.9	6.5	6.1	5.6
A Sum of food values	41.10	52.3	52.6	49.8	47.1	44.3	41.6	38.6
B Whole meal GGE		44.00						
A-B (inaccuracy)		8.3	8.6	5.8	3.1	0.3	−2.4	−5.4
10								
30 g Whole-rye pumpernickel	6.00	7.8	7.7	7.3	6.9	6.5	6.1	5.6
5 g Margarine	0.00	0.0	0.0	0.0	0.0	0.0	0.0	0.0
175 mL Strawberry yoghurt	7.80	10.1	10.0	9.5	8.9	8.4	7.9	7.3
142 g Canned fruit	10.40	13.3	13.3	12.6	11.9	11.2	10.5	9.8
125 mL Orange juice	6.00	7.8	7.7	7.3	6.9	6.5	6.1	5.6
A Sum of food values	30.20	39.0	38.6	36.6	34.6	32.6	30.5	28.4
B Whole meal GGE		34.54						
A-B (inaccuracy)		4.5	4.1	2.1	0.0	−2.0	−4.0	−6.2
11								
100 g Egg	0.00	0.0	0.0	0.0	0.0	0.0	0.0	0.0
75 g French fries	10.90	13.9	13.9	13.2	12.5	11.8	11.0	10.2
25 g While-wheat bread	7.70	10.0	9.8	9.3	8.8	8.3	7.8	7.2
5 g Margarine	0.00	0.0	0.0	0.0	0.0	0.0	0.0	0.0
125 mL Orange juice	6.00	7.8	7.7	7.3	6.9	6.5	6.1	5.6
A Sum of food values	24.60	31.7	31.5	29.8	28.2	26.5	24.9	23.1
B Whole meal GGE		29.06						
A-B (inaccuracy)		2.6	2.4	0.8	−0.9	−2.5	−4.2	−5.9
12								
60 g Bran muffin	15.20	18.9	19.4	18.4	17.4	16.4	15.4	14.3
10 g Margarine	0.00	0.0	0.0	0.0	0.0	0.0	0.0	0.0
125 mL Orange juice	6.00	7.8	7.7	7.3	6.9	6.5	6.1	5.6
A Sum of food values	21.20	26.7	27.1	25.7	24.3	22.9	21.4	19.9
B Whole meal GGE		25.52						
A-B (inaccuracy)		1.2	1.6	0.2	−1.2	−2.7	−4.1	−5.6
13								
175 mL Strawberry yoghurt	7.80	10.1	10.0	9.5	8.9	8.4	7.9	7.3
142 g Canned fruit	10.40	13.3	13.3	12.6	11.9	11.2	10.5	9.8
A Sum of food values	18.20	23.4	23.3	22.1	20.8	19.6	18.4	17.1
B Whole meal GE_50_		22.28						
A-B (inaccuracy)		1.1	1.0	−0.2	−1.4	−2.7	−3.9	−5.2

^1^ GL calculated from carbohydrate × glycaemic index values in International tables of glycaemic index and glycaemic load values: 2002 (Foster-Powell et al.) [15]. ^2^ GGE based on 50 g reference point by adjusting GL for homeostasis using Equation (1). ^3^ B Whole meal GGE calculated by entering the GL sum for the meal into Equation (1).

**Table 4:** Accumulation of inaccuracy as disparities between true (quadratic) and linear estimates of the relative glycaemic impact of meals obtained by adding linear estimates of GGE values for individual foods in a meal compared with GGE for whole meal, and its dependence on glucose reference dose. Meals shown are realistic Australasian non-breakfast meals.

			Linear Estimates of GGE Using Equations in Table 1					
			Glucose Reference					
Meal	GL ^1^	GGE_50_^2^	10	20	30	40	50	60
14								
2 slices white bread (54 g)	20.00	24.2	25.6	24.2	22.9	21.6	20.2	18.8
2 tsp margarine (10 g)	0.00	0.0	0.0	0.0	0.0	0.0	0.0	0.0
1 tablesp honey (21 g)	9.00	11.6	11.5	10.9	10.3	9.7	9.1	8.5
250 mL fruit drink (256 g)	8.00	10.3	10.2	9.7	9.2	8.6	8.1	7.5
A Sum of food values	37.00	46.2	47.3	44.8	42.4	39.9	37.4	34.8
B ^3^ Whole meal GGE		40.63						
A-B (inaccuracy)		5.5	6.7	4.2	1.7	−0.7	−3.2	−5.9
15								
1 cup instant noodles (180 g)	14.00	17.5	17.9	17.0	16.0	15.1	14.2	13.2
1 apple (120 g)	5.00	6.6	6.4	6.1	5.7	5.4	5.1	4.7
1 soft cereal bar (340 g)	19.00	23.2	24.3	23.0	21.8	20.5	19.2	17.9
A Sum of food values	38.00	47.2	48.6	46.1	43.5	41.0	38.4	35.7
B Whole meal GGE		41.47						
A-B (inaccuracy)		5.8	7.1	4.6	2.0	−0.5	−3.1	−5.8
16								
1 roast chicken thigh (119 g)	0.00	0.0	0.0	0.0	0.0	0.0	0.0	0.0
1 baked potato (90 g)	14.00	17.5	17.9	17.0	16.0	15.1	14.2	13.2
Portion roast pumpkin (150 g)	6.00	7.8	7.7	7.3	6.9	6.5	6.1	5.6
1 med. portion sweet potato (70 g)	6.00	7.8	7.7	7.3	6.9	6.5	6.1	5.6
1 corn on cob (100 g)	14.00	17.5	17.9	17.0	16.0	15.1	14.2	13.2
A Sum of food values	40.00	50.7	51.2	48.5	45.8	43.1	40.4	37.6
B Whole meal GGE		43.12						
A-B (inaccuracy)		7.6	8.0	5.4	2.7	0.0	−2.7	−5.5
17								
2 sausages (142 g)	2.00	2.7	2.6	2.4	2.3	2.2	2.0	1.9
1 cup mashed potato (180 g)	22.00	26.4	28.1	26.7	25.2	23.7	22.2	20.7
1 cup green salad (105 g)	0.00	0.0	0.0	0.0	0.0	0.0	0.0	0.0
2 slices canned beetroot (80 g)	4.00	5.3	5.1	4.8	4.6	4.3	4.0	3.8
1 cup canned peaches (208 g)	9.00	11.6	11.5	10.9	10.3	9.7	9.1	8.5
0.5 cup custard (120 g)	7.00	9.1	9.0	8.5	8.0	7.5	7.1	6.6
A Sum of food values	44.00	54.98	56.28	53.33	50.38	47.43	44.48	41.36
B Whole meal GGE		46.25						
A-B (inaccuracy)		8.7	10.0	7.1	4.1	1.2	−1.8	−4.9
18								
1 individual steak pie (172 g)	12.00	15.2	15.3	14.5	13.7	12.9	12.1	11.3
1 cup fried potatoes (80 g)	13.00	16.4	16.6	15.8	14.9	14.0	13.1	12.2
1 apple (120 g)	5.00	6.6	6.4	6.1	5.7	5.4	5.1	4.7
A Sum of food values	30.00	38.12	38.37	36.36	34.35	32.34	30.33	28.20
B Whole meal GGE		34.35						
A-B (inaccuracy)		3.77	4.02	2.01	0.00	−2.01	−4.02	−6.15
19								
1 filled bread roll (77 g)	29.00	33.4	37.1	35.1	33.2	31.3	29.3	27.3
1 banana (120 g)	16.00	19.8	20.5	19.4	18.3	17.2	16.2	15.0
1 can “Coca-cola” (365 g)	23.00	27.4	29.4	27.9	26.3	24.8	23.3	21.6
A Sum of food values	68.00	80.6	87.0	82.4	77.9	73.3	68.7	63.9
B Whole meal GGE		60.55						
A-B (inaccuracy)		20.1	26.4	21.9	17.3	12.8	8.2	3.4
20								
2 medium potatoes (180 g)	30.00	34.4	38.4	36.4	34.4	32.3	30.3	28.2
1 medium steak (145 g)	0.00	0.0	0.0	0.0	0.0	0.0	0.0	0.0
0.5 cups broccoli (82 g)	1.00	1.3	1.3	1.2	1.1	1.1	1.0	0.9
0.5 cups pumpkin (110 g)	4.00	5.3	5.1	4.8	4.6	4.3	4.0	3.8
0.5 cups carrot (800 g)	2.00	2.7	2.6	2.4	2.3	2.2	2.0	1.9
1 cup ice cream (143 g)	12.00	15.2	15.3	14.5	13.7	12.9	12.1	11.3
0.5 cups fruit salad (120 g)	8.00	10.3	10.2	9.7	9.2	8.6	8.1	7.5
A Sum of food values	57.00	69.16	72.90	69.08	65.27	61.45	57.63	53.58
B Whole meal GGE		54.95						
A-B (inaccuracy)		14.21	17.95	14.13	10.32	6.50	2.68	−1.37

^1^ GL calculated from carbohydrate × glycaemic index values in International tables of glycaemic index and glycaemic load values: 2002 (Foster-Powell et al.) [15]. ^2^ GGE based on 50 g glucose reference point by adjusting GL for homeostasis using Equation (1). ^3^ B Whole meal GGE is calculated by entering the GL sum for the meal into Equation (1).

**Table 5:** Effect of total GGE content of meal and number of carbohydrate foods on cumulative inaccuracy, assuming an equal distribution of GGE between foods in a meal.

	Total GGE Content of Meal							
Number of Foods	20 g		30 g		40 g		50 g	
	GGE/Food	CI *	GGE/Food	CI	GGE/Food	CI	GGE/Food	CI
1	20	0.00	30	0.00	40	0.00	50	0.00
2	10	−1.34	15	−3.02	20	−5.36	25	−8.38
3	6.66	−1.78	10	−4.02	13.33	−7.13	16.67	−11.14
4	5.0	−2.01	7.5	−4.52	10	−8.04	12.5	−12.56

* CI = cumulative inaccuracy.

## Data Availability

Not applicable.
